# HDAC6 Deacetylase Activity Is Required for Hypoxia-Induced Invadopodia Formation and Cell Invasion

**DOI:** 10.1371/journal.pone.0055529

**Published:** 2013-02-06

**Authors:** Dominique Arsenault, Karine Brochu-Gaudreau, Martine Charbonneau, Claire M. Dubois

**Affiliations:** Immunology Division, Department of Pediatrics, Faculty of Medicine and Health Sciences, Université de Sherbrooke, Sherbrooke, Québec, Canada; Seoul National University, Republic of Korea

## Abstract

Despite significant progress in the cancer field, tumor cell invasion and metastasis remain a major clinical challenge. Cell invasion across tissue boundaries depends largely on extracellular matrix degradation, which can be initiated by formation of actin-rich cell structures specialized in matrix degradation called invadopodia. Although the hypoxic microenvironment within solid tumors has been increasingly recognized as an important driver of local invasion and metastasis, little is known about how hypoxia influences invadopodia biogenesis. Here, we show that histone deacetylase 6 (HDAC6), a cytoplasmic member of the histone deacetylase family, is a novel modulator of hypoxia-induced invadopodia formation. Hypoxia was found to enhance HDAC6 tubulin deacetylase activity through activation of the EGFR pathway. Activated HDAC6, in turn, triggered Smad3 phosphorylation resulting in nuclear accumulation. Inhibition of HDAC6 activity or knockdown of the protein inhibited both hypoxia-induced Smad3 activation and invadopodia formation. Our data provide evidence that hypoxia influences invadopodia formation in a biphasic manner, which involves the activation of HDAC6 deacetylase activity by EGFR, resulting in enhanced Smad phosphorylation and nuclear accumulation. The identification of HDAC6 as a key participant of hypoxia-induced cell invasion may have important therapeutic implications for the treatment of metastasis in cancer patients.

## Introduction

Hypoxia is a hallmark of solid tumor that leads to cell invasion and metastasis, which are the major causes of cancer patient death. Through a complex series of events, hypoxia enhances the propensity of cancer cell to invade. Hypoxia-inducible factor-1 (HIF-1) transcriptional activity was proposed to be in part responsible for the enhanced invasive properties of cancer cell through the regulation of a variety of extracellular proteases, growth factors and receptors [1], [2]. Among these, TGFβ, a well-known cytokine involved in many steps of cancer dissemination, has been shown to be increased in cancer cells, Indeed, levels of active TGFβ as well as activation of the Smad-dependent signaling pathway have been shown to be augmented under hypoxia through increased processing of the precursor from and/or bioactivation of the latent growth factor [3]–[5]. Besides transcriptional events regulated by hypoxia, recent data support a role of post-transcriptional mechanisms. For example, strategic relocalization of proteases, integrins and growth factor receptors at recycling endosomes/cell surface and/or increased phosphorylation/activation of kinases was shown to contribute to enhanced invasiveness of cancer cells [6]–[10]. Despite the progress in understanding how hypoxia regulates cell invasion, the exact mechanisms involved remain poorly defined.

Recent reports have highlighted the implication of histone deacetylases (HDACs) in tumor progression and invasion and their potential use as therapeutic targets for cancer therapy is now emerging [11], [12]. Eleven classical HDAC family members have been identified in human. These enzymes are mainly involved in deacetylation of histones [13], [14], although a growing list of non-histone proteins such as tubulin, cortactin, heat shock proteins and catenin have been shown to be HDAC substrates [15]. Because these substrates as well as abnormal activation and deactivation of transcription based on histone status have been associated with tumorigenesis, various pan-specific HDAC inhibitors have been evaluated in clinical trials for cancer therapy [16]–[18]. Data showed that these inhibitors caused numerous side-effects and had poor anti-cancer activity against solid tumors [19]–[21], possibly due to the role of HDACs as post-translational modifiers of numerous key proteins [22]. These results fostered the study of the role of individual HDAC enzymes in cancer progression. Among the HDACs family members, HDAC6 is a cytoplasmic resident protein that is overexpressed in many cancers [23]–[26] and has been implicated in cell migration and invasion [27]–[29]. Alpha-tubulin is the major substrate of HDAC6 but emerging evidence indicates that cortactin can also be deacetylated by HDAC6 [29]–[31]. These cytoskeleton components play various roles in cell division, protein trafficking, cell motility, and signal transduction [32]. Recently, HDAC6 has been shown to be a key regulator during epithelial-to-mesenchymal transition (EMT) by enhancing Smad3 activation and nuclear translocation possibly through desequestration of Smad3 from acetylated α-tubulin [27], [33]. HDAC6 has been shown to be an estrogen-regulated gene [34] but growth factors such as TGFβ and EGF can also enhance its activity *in vitro* [27], [35]. These studies suggested the participation of HDAC6 in cancer cell invasion and metastasis, possibly through a mechanism involving TGFβ action/signaling.

During the process of metastasis, tumor cells must degrade extracellular matrix (ECM) in order to migrate through blood vessels and colonize distant sites. To achieve tissue invasion, cancer cells initiate a number of developmental processes. Acquisition of migratory and invasive properties involves reorganization of the actin cytoskeleton and concomitant formation of invadopodia. Invadopodia are dynamic extensions of the plasma membrane enriched in F-actin as well as cortactin, a key signaling protein involved in many cellular processes, including cell adhesion, endocytosis/exocytosis, migration and actin-driven membrane protrusion formation. A number of signaling events, mostly involving tyrosine phosphorylation, occur within invadopodes and they cooperate to up-regulate expression of integrins, metalloproteases and other components involved in invadopodia formation and focal ECM degradation [36]–[41]. Whereas there is relatively little known concerning environmental factors that stimulate invadopodia formation, growth factors such as TGFβ and EGF, acidic pH, and ECM-induced integrin activation have been shown to trigger invadopodia formation [41]–[45]. In this respect, we have recently shown that hypoxia and acidosis increased invadopodia formation through activation/phosphorylation of p90 ribosomal S6 kinase (p90RSK) [10].

In this report, we present data showing that hypoxia enhances invadopodia formation and cell invasion through the enhancement of HDAC6 deacetylase activity. Furthermore, we show that this event is regulated through activation of the EGF receptor. The enhanced activity of HDAC6 allowed Smad3 activation and nuclear translocation, which was involved in hypoxia-induced invadopodia production. These findings provide new insights into the molecular mechanism linking the tumor hypoxic microenvironment and cell invasion and open the way to further investigations aimed at targeting HDAC6 for the treatment of metastasis.

## Materials and Methods

### Antibodies and Reagents

Antibodies used for immunofluorescence (IF) microscopy and Western blotting (WB) were used at the following dilutions: mouse anti-cortactin (IF 1∶250) and rabbit anti-phospho-smad3 (IF 1∶200) antibodies were purchased from Millipore (Billerica, MA), rabbit anti-smad3 (IF 1∶200), rabbit anti-EGFR (WB 1∶1000) and rabbit anti-phospho-tyrosine (WB 1∶1000) antibodies were purchased from Cell Signaling Technology (Danvers, MA), rabbit anti-HDAC6 antibody (IF 1∶200, WB 1∶1000) was bought from Santa Cruz Biotechnology (Santa Crux, CA), mouse anti-α-tubulin (IF 1∶1000, WB 1∶1000) and mouse anti-acetylated-tubulin (IF 1∶200, WB 1∶1000) antibodies were from Sigma-Aldrich (St-Louis, MO), Oregon-488 conjugated phalloïdin, 4′,6-diamidino-2-phenylindole dilactate (DAPI) were from Molecular Probes (Eugene, OR). All secondary antibodies used for IF were from Molecular Probes (Eugene, OR). Secondary anti-mouse-conjugated horseradish peroxidase (HRP) and anti-rabbit-conjugated HRP were obtained from Amersham Biosciences (Baie d’Urfé, QC). The following reagents were used at the indicated concentrations: TGFβ (2 ng/mL, 5 ng/mL) from PerproTech Inc. (Rocky Hill, NJ), Tyrphostin AG-1478 (2 µM, 10 µM) from BioMol International (Plymouth, PA), SIS3 (0.5 µM, 5.0 µM) from Calbiochem International (La Jolla, CA), Trichostatin A (2.5 µM, 5.0 µM) and Ly364947 (50 nM, 500 nM) from Sigma-Aldrich. Tubacin (10 µM) was purchase from Sigma-Aldrich and Nitubacin (10 µM) was a generous gift of Dr. Ralph L. Schreiber (Center for System Biology, Massachusetts General Hospital, Boston, MA).

### Plasmids

pGEM7ZF-hfur was a generous gift of Dr. Gary Thomas (University of Oregon, Vollum Institute, Portland, OR). pGEM7ZF-hfur was digested with EcoRI and EcoICRI and inserted into pcDNA3 (Promega, Madison, WI) digested with EcoRI and EcoRV. AT-PDX gene, kindly provided by Dr. Gary Thomas was inserted into the EcoRI/ApaI cloning site of pcDNA3 plasmid vector. shRNA against HDAC6 or scramble shRNA (control) were from SABiosciences (Frederick, MD).

### Cell Culture and Transfection

Human fibrosarcoma HT-1080 cells were obtained from the American Type Culture Collection (ATCC, Rockville, MD). Cells were cultured in complete media consisting of minimal essential medium (Invitrogen, Carlsbad, CA) supplemented with 10% fetal bovine serum. HT-1080 cells were stably transfected with pcDNA3-hFur, pcDNA3-PDX and HDAC6 shRNA using the TransIT®-LT1 Transfection Reagent (Mirus, Madison, WI), according to the manufacturer’s instructions. pcDNA3-hFur and pcDNA3-PDX stably transfected cells were maintained in complete media containing G-418 (600 µg/ml). HDAC6shRNA transfected cells were maintained in complete media containing puromycin (2 µg/mL) (InvivoGen, San Diego, CA). Hypoxic conditions were used as follows. Cells were serum-starved and placed in an Invivo2 400 hypoxic workstation maintained at 1% O_2_, 5% CO2 compensated with N2 for different periods of time, as indicated in the legends of the Figures.

### Immunofluorescence Staining

Cells were seeded at a density of 5 X 10^4^ cells/cm^2^ in circular 15-mm diameter glass coverslips coated with gelatin (0.2%). The coverslips were placed in 12-well culture plates and incubated for 24 h. The cells were serum-starved for 3 h prior to exposure to normoxic or hypoxic conditions for different periods of time. All reagents were added 30 min prior to cell exposure to experimental conditions. Following incubations, the cells were washed with PBS, fixed with 2% paraformaldehyde (30 min, 4°C) permeabilized (0.1% Triton-X100), and immunostained for 2 h with primary antibody or stained with phalloïdin for 45 min. The cells were then washed and incubated with Alexa–conjugated secondary antibodies at 4°C for 1 h. As negative controls, species- and isotype-matched immunoglobulin G were used instead of primary antibody.

### Invadopodia Assay

Coverslips were prepared as described [46] using Oregon Green gelatin (Invitrogen). Thirty thousand cells were seeded on each coverslip, allowed to adhere, and incubated in MEM containing 0.5% FBS. Following various incubation times, as indicated in the legends of the Figures, cells were fixed with 1% paraformaldehyde for 30 min at 4°C and stained with DAPI for 5 min at room temperature. Cells were visualized by fluorescence microscopy, and cells forming invadopodia were counted. Invadopodia were identified by the areas of matrix degradation characterized by loss of green fluorescence. A minimum of three hundred cells were counted per coverslip.

### Confocal Microscopy

Cells were examined with a scanning confocal microscope (FV1000, Olympus, Tokyo, Japan) equipped with an inverted microscope and a 63× oil immersion objective (Olympus). Specimens were laser-excited at 488 nm, 563 nm and 405 nm. Serial horizontal optical sections of 512×512 pixels with 2-times line averaging were taken at 0.11 µm intervals through the entire thickness of the cell (optical resolution: lateral - 0.18 µm; axial - 0.25 µm). Images were acquired on the same day, typically from 10 cells of similar size from each experimental condition, under identical settings.

### Image Analysis and Quantitative Measurements

In the case of Alexa-488/DAPI- or Alexa-546/DAPI-merged fluorescence images, dot fluorograms were obtained by plotting pixel values of each marker in the vertical and horizontal axes, respectively. Thresholds were determined using single markers and noise and background were subtracted. Quadrant markers were adjusted forming background (C), red- or green-only (D), blue-only (A) and co-localization areas (B). The percentage of colocalization was calculated as follows (B)/((B)+(A)) x 100. To determine fluorescence quantification, images were processed for a total of 35 slices per cell on 10 size-matched cells for each experimental condition, and experiments were performed at least three times. Images were shown in pseudo-color, according to their original fluorochromes, merged (FluoView software (Olympus), then cropped and assembled (Adobe Photoshop software, Adobe Systems, Mountain View, CA). To quantify the areas of degradation, pictures of fluorescent gelatin were acquired and captured into ImagePro imaging software (Media Cybernetics Inc, Bethesda, MD) and degradation areas were calculated in pixel intensities for a total of at least 20 cells per coverslip.

### Western Blots

Cells (1×10^6^ per 100-mm petri dishes) were incubated overnight and serum-starved for 4 h prior to exposure to normoxic (21% O2) or hypoxic (1% O2) conditions. The cells were washed in ice-cold PBS and lysed with a M-RIPA lysis buffer (50 mM Tris-HCl pH 7.4, 150 mM NaCl, 0.1% Na-deoxycholate, 8 mM EDTA pH 8.0, 10% NP-40 and complete mini EDTA-free protease inhibitor cocktail tablets (Roche, Indianapolis, IN). Immunoblotting was performed as described [47].

### Immunoprecipitation

Cells (2×10^7^ per 150-mm petri dishes) were incubated overnight and serum-starved 3 to 4 h prior to stimulation under normoxic (21% O_2_) or hypoxic (1% O_2_) conditions for 30, 60 and 120 min. Cells were then washed with ice-cold PBS and lysed with NP-40 phosphate buffer (1% NP-40, 150 mM NaCl, 10 mM sodium phosphate, complete mini EDTA-free protease inhibitor cocktail tablet (Roche Applied Science). One mg total protein was immunoprecipitated with anti-EGFR antibody (1∶100) and blotted with anti-phospho-tyrosine antibody (1∶1000) as described in the Western Blot section.

### Three-dimensional Invasion Assays

Collagen type I matrix was prepared as follows, Aliquots (50 µL) of agarose containing 10% FBS were deposited in 96 well culture plates. Aliquots (50 µL) of fibrillar type I collagen (3 mg/mL) (R&D Systems, Minneapolis, MN) were then prepared following the manufacturer’s instructions and layered on top of the agarose layer. Cells (2×10^4^/100 µl of MEM) were serum-starved overnight, seeded on top of the collagen layer and incubated for 24 h under normoxic or hypoxic conditions. The cells were labeled with CellTraceTM Calcein Green AM (Invitrogen) 1 h prior to the end of incubation. Cells were then washed with PBS and fixed with 3% glutaraldehyde for 30 min followed by confocal microscopy analysis using a FV1000 Olympus confocal microscope. Collagen matrix pellets were scanned along the Z-axis. Cells that had invaded the collagen gel were imaged and quantitated at each 5 µm layers within the gel. Cell invasion was expressed as a ratio of the fluorescence intensity of each 5 µm layer/ fluorescence intensity of the top 5 µm layer (non-invading cells).

### Statistical Analysis

Unpaired Student’s *t*-test was used to assess statistical significance. A *p* value < 0.05 was considered to be significant.

## Results

### Hypoxia Enhances Invadopodia Formation and ECM Degradation Through the TGFβ/Smad3-dependent Signaling Pathway

**Figure 1:**
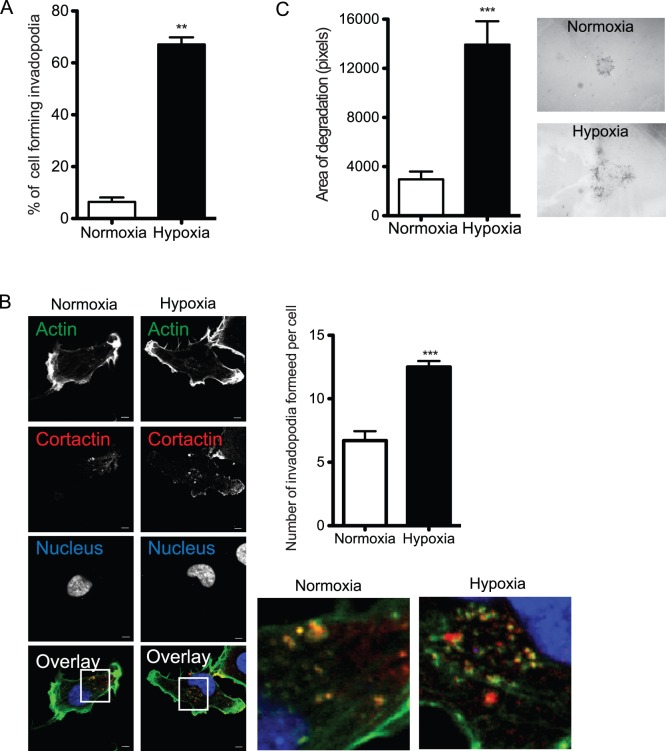
Hypoxia promotes invadopodia formation. HT-1080 cells were cultured on FITC-gelatin-coated slides in normoxia or hypoxia for 4 h (**B**) or 10 h (**A, C**). (**A**) Percentage of cells forming invadopodia. (**B**) Micrographs of actin (green), cortactin (red) nucleus (blue) and merged images are shown. The associated graph shows the number of F-actin-positive and cortactin-positive invadopodia per cell as described under Materials and Methods. (**C**) Quantification of ECM degradation (area/cell). The associated micrographs show representative ECM degradation area for a single cell. Columns correspond to the mean ± SEM; ** p < 0.01, *** p < 0.001; scale bars correspond to 5 µm.

Exposure of HT-1080 human fibrosarcoma cells to hypoxic conditions (1% O_2_) for 10 h resulted in a 10-fold increase in the percentage of cells forming invadopodia compared to normoxic conditions (21% O_2_) (Figure 1A). Quantification of the number of invadopodia identified by the colocalization of actin and cortactin, two known markers of invadopodia [36], as well as the extend of ECM degradation per cells showed a significant increase in hypoxic cells compared to their normoxic counterpart (Figures 1B, 1C). Similarly to the data recently published by our group [10], these observations showed that hypoxia enhanced both the formation of invadopodia and their capacity to degrade ECM.

**Figure 2:**
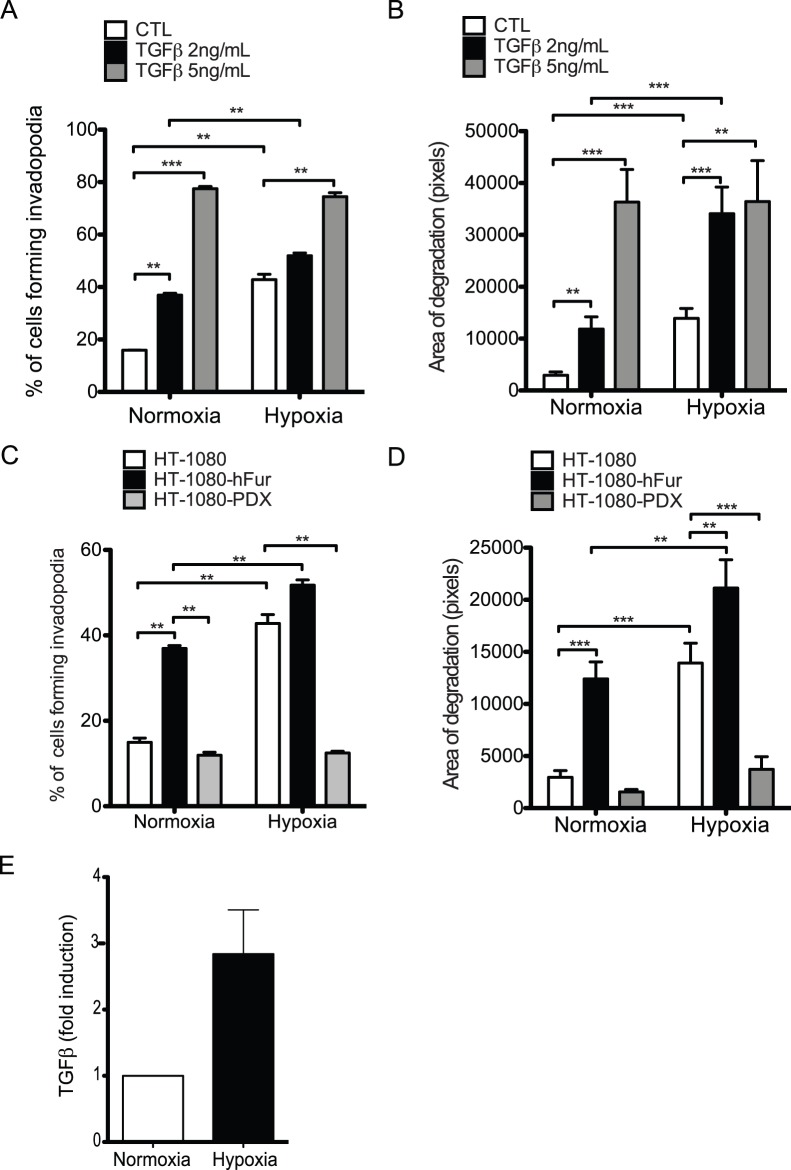
Endogenous TGFβ is involved in hypoxia-induced invadopodia formation. (**A**, **B**) HT-1080 cells were cultured on FITC-gelatin-coated slides in normoxia or hypoxia for 10 h in the presence or absence of TGFβ added at the indicated concentrations. (**A**) Percentage of cells forming invadopodia. (**B**) Quantification of ECM degradation (area/cell). (**C**, **D**) HT-1080, HT-1080-hFur and HT-1080-PDX cells were cultured on FITC-gelatin-coated slides in normoxia or hypoxia for 10 h. (**C**) Percentage of cells forming invadopodia. (**D**) Quantification of ECM degradation (area/cell). (**E**) Total TGFβ was quantitated in supernatants of cells cultured in normoxia or hypoxia for 24 h. Columns correspond to the mean ± SEM; ** p < 0.01, *** p < 0.005.

We have previously reported that hypoxia induced an increase in pro-TGFβ maturation through up-regulation of the proprotein convertase furin [2]. In addition, TGFβ was shown to promote the invasion of cancer cells [48]–[50] and the formation of F-actin cores in CA1D human breast cancer cells [45] and podosomes in endothelial cells [44]. To address the potential role of TGFβ in hypoxia-induced invadopodia formation, we first added TGFβ to HT-1080 cells incubated under normoxic or hypoxic conditions. Low concentrations of TGFβ increased invadopodia formation and ECM degradation in normoxic cells (Figures 2A, 2B). Alternatively, higher concentrations of TGFβ enhanced invadopodia formation and ECM degradation in both normoxic and hypoxic cells as well as the number of invadopodia formed per cell (Figures 2A, B). These results indicated that TGFβ mimicked the impact of hypoxia on invadopodia formation and function presumably due to the capacity of hypoxia to increase TGFβ processing/production [2]. TGFβ is therefore a candidate mediator by which invadopodia are stimulated by hypoxia.

To investigate the implication of endogenous TGFβ in hypoxia-induced invadopodia formation, we took advantage of the ability of the proprotein convertase furin to recognize and cleave at the basic sequences found in TGFβ1,-2 and -3 maturation site, providing an efficient way to interfere with activation of TGFβ [51]. Overexpression of furin in HT-1080 cells led to a 2-fold increase in the percentage of cells forming invadopodia in normoxic cells. In contrast, overexpression of α1-antitrypsin-Portland (α1-PDX), a modified serpin with potent furin inhibitory activity [52], [53] did not modify the capacity of the cells to produce invadopodia under normoxic conditions while it completely blunted invadopodia formation and ECM degradation in cells incubated under hypoxia (Figures 2C, D). Furthermore, the levels of bioactive TGFβ1, as measured using ELISA assays, were 2.8-fold higher in supernatants from hypoxic cells compared to supernatants from non-hypoxic cells (Figure 2E). Taken together, these results suggested that endogenous TGFβ was involved in hypoxia-induced invadopodia formation and ECM degradation.

**Figure 3:**
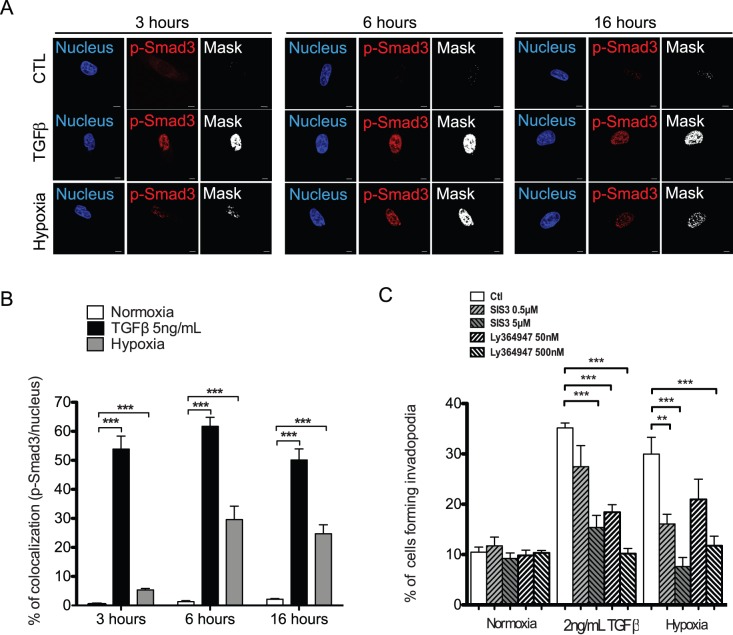
The Smad3-dependent signaling pathway is involved in hypoxia-induced invadopodia formation. (**A, B**) HT-1080 cells were seeded on gelatin-coated slides and incubated in the presence or absence of TGFβ or in hypoxia for 3, 6 or 16 h. Cells were labeled for p-Smad3 and nucleus (DAPI) and analyzed by confocal microscopy. (**A**) Micrographs of p-Smad3 (red), nucleus (blue) and merged images (binary mask-overlay). (**B**) Graph showing the percentage of colocalization of p-Smad3 with nucleus measured as described under Materials and Methods. (**C**) Cells were seeded on FITC-gelatin-coated slides, preincubated in the presence or absence of SIS3 or Ly364947 for 30 min and incubated in normoxia in the presence or absence of TGFβ or hypoxia for 10 h. The graph shows the percentage of cells forming invadopodia. Columns correspond to the mean ± SEM; ** p = 0.01, *** p < 0.001; Scale bars correspond to 5 µm.

TGFβ signals, in part, through a canonical pathway involving Smad2/3 and Smad4 that forms a multimeric Smad complex which induces or represses TGFβ responsive genes once accumulated in the nucleus [54]. Recently, it has been shown that TGFβ induced invadopodia production by enhancing MMP-9 production, an event that was associated with Smad-dependent signaling pathway [45]. In addition, hypoxia was shown to induce the expression of various genes through cooperation between Smad3 and HIF-1/-2 [55], [56]. To further investigate the role of TGFβ in hypoxia-induced invadopodia, we first tested whether exposure to hypoxia led to Smad2 and Smad3 activation and nuclear accumulation using antibodies that detect the activated (phosphorylation on C-terminal serine residues) state of these Smads. TGFβ stimulations were used as controls. Results revealed that hypoxia increased the levels of Smad3 phosphorylation (Figure S1) and the accumulation of p-Smad3 in the nucleus of HT-1080 cells, with a maximal effect at 6 h (Figures 3A, 3B). In contrast, Smad 2 phosphorylation or nuclear accumulation was only marginally affected by hypoxia (Figures S1 and S2). Stimulation of the cells with TGFβ under normoxic conditions resulted in a significant increase in nuclear accumulation of both p-Smad2 (Figure S2) and p-Smad3 (Figures 3A, 3B). Next, we used pharmacological inhibitors to investigate the role of the Smad3-dependent signaling pathway in hypoxia-induced invadopodia formation. Treatment of HT-1080 cells with either Ly364947, a selective inhibitor of TGFβR1 [57], or SIS3, a cell-permeable selective inhibitor of TGFβ-dependent Smad3 phosphorylation and Smad3-mediated cellular signaling [58], had no significant impact on the percentage of cells forming invadopodia under normoxic conditions (Figure 3C). This result correlated with the previous observation that Smad3 was not activated nor accumulated in the nucleus of normoxic cells. Conversely, the addition of Ly364947 or SIS3 to cells cultured under hypoxic conditions resulted in a concentration-dependent decrease in the percentage of cells forming invadopodia with a complete inhibition observed at the highest concentration (Figure 3C). Taken together, these results strongly suggested that TGFβ played a critical role in hypoxia-induced invadopodia formation in HT-1080 cells through the Smad3-dependent signaling pathway.

### HDAC6 Activity is Required for Hypoxia-induced Smad3 Nuclear Translocation and Invadopodia Formation

**Figure 4:**
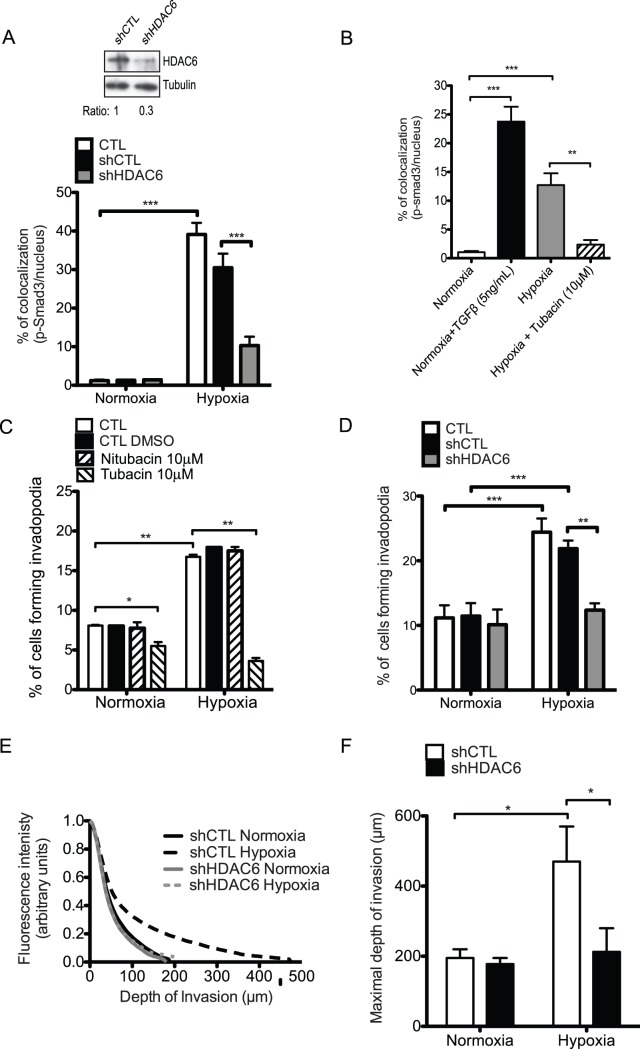
HDAC6 is involved in hypoxia-induced invadopodia formation. (**A)** HT-1080 (CTL), HT-1080 transfected with control shRNA (sh-CTL) or shRNA targeting HDAC6 (shHDAC6) or (**B**) untransfected HT-1080 cells incubated in the presence or absence of Tubacin or TGFβ were cultured on gelatin-coated slides in normoxia or hypoxia for 6 h. (**A, B**) Graph showing the percentage of colocalization of p-Smad3 with nucleus (DAPI) measured as described under Materials and Methods. (**A**) Immunoblot showing inhibition of HDAC6 expression by HDAC6 shRNA. (**C**) HT-1080 cells were cultured on FITC-gelatin-coated slides in normoxia or hypoxia for 10 h and treated with DMSO (vehicle CTL), Tubacin or Nitubacin (Negative CTL). The graph shows the percentage of cells forming invadopodia. (**D-F**) HT-1080 (CTL), HT-1080 transfected with shCTL (sh-CTL) and HT-1080 transfected with shHDAC6 (shHDAC6) were cultured on FITC-gelatin-coated slides (D) or allowed to invade collagen gels (E, F) in normoxia or hypoxia. (**D**) Percentage of cells forming invadopodia for cells incubated for 10 h. (**E, F**) Cells were allowed to invade collagen gels for 24 h and stained as described under Materials and Methods. (**E)** Relative fluorescence intensity of the cells according to the depth of invasion. The arbitrary index of invasion was calculated as described under Materials and Methods. (**F**) Maximal depth of invasion. Columns correspond to the mean ± SEM; * p < 0.04; ** p < 0.002, *** p < 0.001.

HDAC6 has been shown to be involved in microtubule stability through the regulation of the levels of tubulin acetylation [59]. Moreover, recent studies have indicated that HDAC6 was required for TGFβ1-induced EMT, an event that was correlated with HDAC6-induced deacetylation of tubulin and activation of Smad3 [27]. This finding prompted us to investigate the relationship between HDAC6 and Smad3 signaling in hypoxia-induced invadopodia formation. To define the role of HDAC6 in hypoxia-induced Smad3 nuclear accumulation, HT-1080 cells were transfected with HDAC6-targeted shRNA or control shRNA. Knockdown or inhibition of HDAC6 activity with tubacin, a specific inhibitor of HDAC6 tubulin deacetylase activity [60], resulted in a strong inhibition of hypoxia-induced nuclear localization of p-Smad3 compared to an absence of significant reduction in cells transfected with control shRNA (Figure 4A, B). Immunoblotting results confirmed the efficiency of HDAC6 knockdown (Figure 4A). We therefore concluded that HDAC6 activity was required for hypoxia-induced nuclear accumulation of Smad3. To determine whether HDAC6 and HDAC6 deacetylase activity were involved in hypoxia-induced invadopodia formation, HT-1080 cells were transfected with HDAC6-specific shRNA or incubated with tubacin, or its non-functional structural analog nitubacin [60], during invadopodia assays. Knockdown of HDAC6 with shRNA completely inhibited hypoxia-induced invadopodia formation without affecting the normoxic levels of invadopodia (Figure 4D). In addition, tubacin decreased the percentage of cells forming invadopodia in normoxia while completely abolishing invadopodia formation in response to hypoxia (Figure 4C). In contrast, nitubacin treatment did not affect the percentage of invadopodia formed by cells cultured under normoxic or hypoxic conditions (Figure 4C). Similar inhibition was observed when cells were incubated with trichostatin A (TSA), a large spectrum HDAC inhibitor, during the invadopodia assay (Figure S3A). In contrast, valproid acid (VPA), a class I HDAC inhibitor that inhibits HDAC 1,2,3 and 8, failed to reduce the levels of hypoxia-induced invadopodia formation further suggesting that among the HDAC family members HDAC6 is a key mediator of invadopodia formation in hypoxia (Figure S3B). To further assess the relevance of HDAC6 in hypoxia-induced cell invasion, we performed three-dimensional invasion assays. HT-1080 cells, transfected with control or HDAC6 shRNAs, were seeded on top of type I collagen matrix and incubated under normoxic or hypoxic conditions for 48 h. Cells that invaded the collagen gels were imaged and quantitated using confocal microscopy. Results showed that HDAC6 knockdown cells lost the ability to deeply invade collagen gels in response to hypoxia, without changing their invasive capacity under normoxic conditions (Figures 4E, 4F). These results indicated an essential role for HDAC6 and HDAC6 deacetylase activity in hypoxia-induced Smad3 activation and cell invasion. They also suggested that HDAC6 levels and/or activity were regulated by hypoxia.

### Hypoxia-induced HDAC6 Activity Involves the EGFR but not the TGFβ Pathway

**Figure 5:**
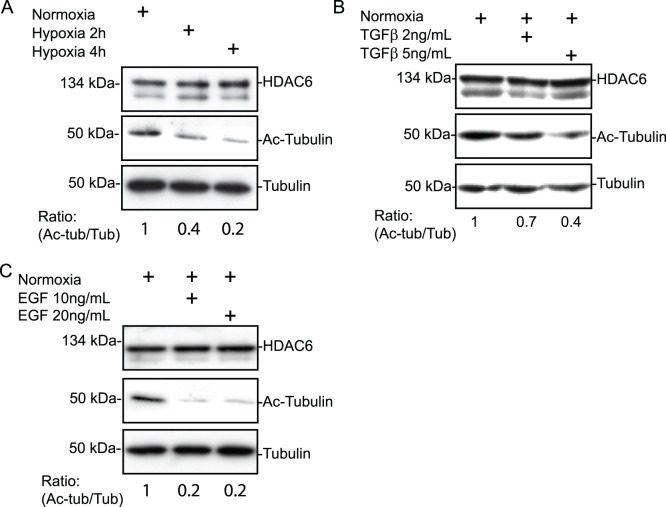
Hypoxia induces HDAC6 deacetylase activity. (**A**) HT-1080 cells were seeded on gelatin-coated slides and incubated in normoxia or hypoxia for 2 h or 4 h and cell lysates immunoblotted for HDAC6, acetylated-tubulin, and tubulin. (B, C) Cells seeded on gelatin-coated slides were incubated in normoxia for 4 h in the presence or absence of (**B**) TGFβ or (**C**) EGF at the indicated concentrations. Immunoblots of HDAC6, acetylated-tubulin, and tubulin.

To define whether hypoxia enhances HDAC6 protein level and/or activity, HT-1080 cells were incubated under normoxic or hypoxic conditions and HDAC6 levels and activity were measured by immunoblotting for HDAC and α-tubulin, respectively. Computation of the ratio of acetylated to total α-tubulin showed that exposure of the cells to hypoxia for 2 and 4 h resulted in a 60% and 80% increase in HDAC6 activity (as visualized by the loss in acetylated tubulin), respectively, without significant modulation in HDAC6 protein levels (Figure 5A). In addition, the levels of HDAC6 activity returned to control (normoxic) conditions upon incubation of the cells for longer periods of time (6–24 h; data not shown).

**Figure 6:**
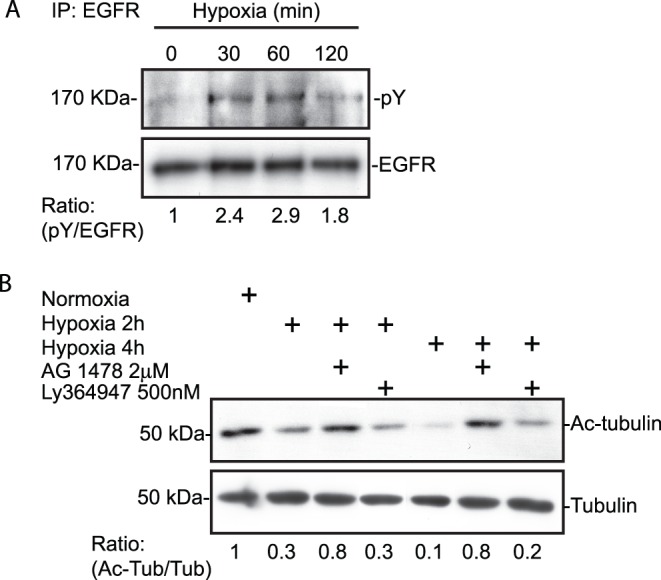
The EGFR signaling pathway is involved in hypoxia-induced HDAC6 activation. (**A**) HT-1080 cells were seeded on gelatin-coated slides and incubated in normoxia or hypoxia for the indicated times. Cell lysates were immunoprecipitated (IP) with anti-EGFR antibodies and immunoblotted with anti-phosphotyrosine and anti-EGFR antibodies. (**B**) HT-1080 cells were seeded on gelatin-coated slides and incubated in normoxia or hypoxia for 2 h and 4 h in the presence or absence of Tyrphostin AG1478 or Ly364947 at the indicated concentration. Cell lysates were immunoblotted with acetylated-tubulin and tubulin antibodies.

Because HDAC6 activity, but not its expression, was affected by hypoxia, we next searched for potential mediators of HDAC6 activation. TGFβ, through the canonical pathway, and EGF, through activation of the EGFR, are among the few stimuli reported to directly modulate HDAC6 activity [27], [35]. Therefore these pathways represent candidate mediators of HDAC6 activation under hypoxic conditions. To define whether TGFβ or EGF influenced HDAC6 tubulin deacetylase activity, HT-1080 cells cultured under normoxic conditions, were incubated for 4 h in the presence or absence of EGF or TGFβ. The addition of EGF or TGFβ resulted in a significant increase in HDAC6 activity as illustrated by the reduction in the levels of acetylated-tubulin over tubulin, whereas HDAC6 protein levels were not altered by either EGF or TGFβ (Figures 5B, 5C). Next, to define whether hypoxia induced the activation of the EGF receptor, HT-1080 cells were incubated for different periods of time under hypoxic conditions. EGFR was then immunoprecipitated followed by immunoblotting with anti-phosphotyrosine antibodies or anti-EGFR antibodies. Results indicated that hypoxia increased EGFR phosphorylation on tyrosine residues with maximal effect at 60 min, although total levels of EGFR remained unchanged (Figure 6A). In addition, we could not detect any significant amounts of EGF in supernatants of cells incubated under normoxic or hypoxic conditions. Next, to investigate the implication of endogenous growth factors in hypoxia-induced HDAC6 activation, cells were pre-treated with either Tyrphostin AG-1478, or Ly364947 to selectively block kinase activity associated with EGFR or TGFβR1, respectively, followed by incubation under hypoxic conditions for 2 or 4 h. Inhibition of EGFR kinase activity almost completely prevented hypoxia-induced HDAC6 deacetylase activity (Figure 6B). In contrast, inhibition of the TGFβR1 kinase had no significant effect (Figure 6B). These data suggested that although EGFR and TGFβR1 are activated under hypoxic conditions, the ability of hypoxia to activate HDAC6 activity is likely to be mediated by EGFR.

**Figure 7:**
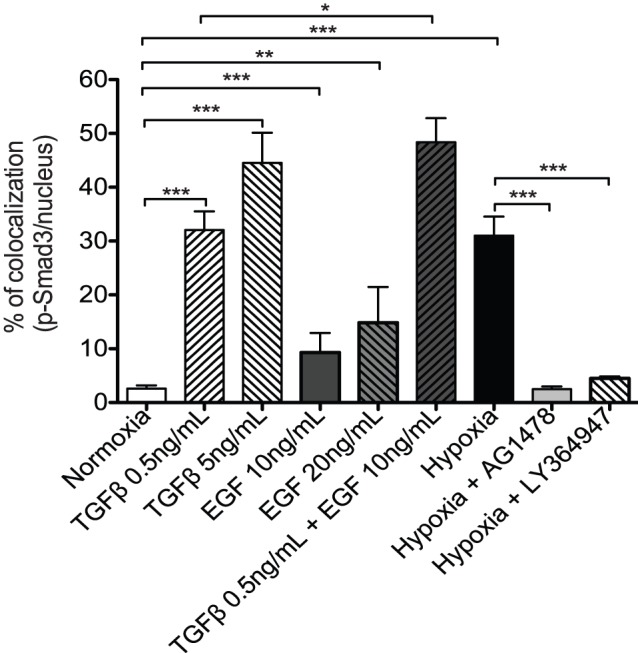
EGF enhances TGFβ signaling and Smad3 nuclear accumulation. HT-1080 cells were seeded on gelatin coated-slides and incubated in normoxia or hypoxia for 6 h in the presence or absence of TGFβ EGF, Tyrphostin AG1478 (2 µM) and Ly364947 (500 nM). Percentage of colocalization of p-Smad3 with nucleus measured as described under Materials and Methods.

Results presented above suggested a relationship between the EGF and TGFβ signaling pathway in hypoxia-induced Smad3 activation and nuclear accumulation. To explore this possibility further, HT-1080 cells were incubated for 6 h under normoxic conditions in the presence of low concentrations of TGFβ with or without EGF, or incubated in hypoxia in the presence or absence of Tyrphostin AG-1478. A low concentration (0.5 ng/mL) of TGFβ was sufficient to significantly induce pSmad3 nuclear accumulation, whereas a higher concentration (5 ng/mL) led to a further increase in pSmad3 nuclear accumulation (Figure 7). Interestingly, EGF alone induced a small but significant accumulation of Smad3 in the nucleus, and addition of 0.5 ng/ml of TGFβ resulted in an additive effect on p-Smad3 nuclear accumulation. Furthermore, similar to results found using TGFβR1 or Smad3 inhibitors (Figure 3C), selective inhibition of EGFR kinase activity with Tyrphostin AG-1478 or TGFβR1 with LY364947 completely abrogated hypoxia-induced nuclear accumulation of p-Smad3 (Figure 7). Taken together, these results suggested that the EGFR and TGFβ signaling pathways cooperated for the nuclear accumulation of pSmad3 under hypoxic conditions.

## Discussion

**Figure 8:**
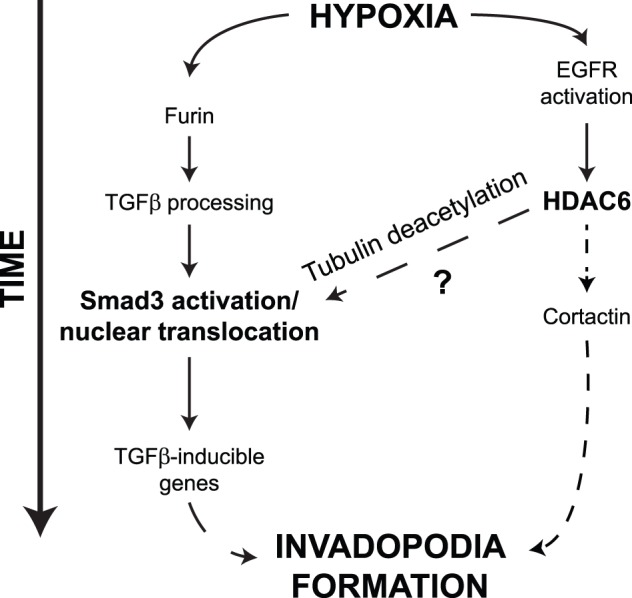
Proposed mechanisms involved in invadopodia formation under hypoxia. The hypoxic microenvironment in solid tumor is proposed to influence invadopodia formation through a biphasic mechanism involving the regulation of HDAC6, followed by activation of the TGFβ/Smad3-dependent signaling pathway. First, hypoxia leads to the activation of the EGF/EGFR pathway, which enhances HDAC6 tubulin deacetylase activity. In parallel, hypoxia increases the expression of the proprotein convertase furin, resulting in enhanced TGFβ processing and bioactivation. HDAC6 tubulin deacetylation may allow the desequestration of Smad3 from the MT network leading to its activation and nuclear translocation. Smad3 translocation leads to enhanced transcription of TGFβ inducible genes and invadopodia production. In this model, HDAC6 might also translocate to the cell periphery where it was shown to be involved in cortactin deacetylation [31] an event needed for the formation of actin-rich invadopodia structures.

In this study, we showed that exposure of fibrosarcoma cells to hypoxia promoted HDAC6 tubulin deacetylase activity that orchestrated Smad3 activation and nuclear translocation. We identified TGFβ and Smad3 signaling pathway as important mediators of hypoxia-induced invadopodia formation. In addition, we demonstrated that the inhibition of HDAC6 expression or deacetylase activity impaired Smad3 activation, an event involved in invadopodia formation and cell invasion triggered by hypoxia. We further provided evidence that hypoxia modulated HDAC6 activity through the activation of EGFR. These results led us to propose that hypoxia promotes invadopodia formation through an EGFR-HDAC6-TGFβ/Smad3 axis (Figure 8).

TGFβ is a secreted growth factor that controls numerous biological processes such as proliferation and differentiation. It is synthesized as a precursor protein that requires proteolytic cleavage by proprotein convertases in order to become biologically active [51], [61]. TGFβ is up-regulated in various types of cancers [62]–[64] and has been shown to be involved in invadopodia formation [45]. Accordingly, our results support a role for TGFβ in hypoxia-induced invadopodia formation. Several reports have shown that hypoxia leads to the bioactivation of latent TGFβ [3], [65] and we have previously shown that such increase in TGFβ bioactivation occurred, at least in part, through up-regulation of the proprotein convertase furin [2]. Our data indicated that hypoxia-induced invadopodia formation was mimicked by overexpression of furin, whereas it was reverted by either inhibition of the endogenous convertase or inhibition of TGFβ signaling strongly suggesting that enhanced bioactivation of endogenous TGFβ is one of the mechanism involved in invadopodia formation induced by hypoxia. Because furin was also shown to process other substrates involved in invadopodia formation, such as the metalloproteinase MT1-MMP [66], [67], one cannot rule out the participation of other furin substrates in invadopodia formation/function.

Canonical TGFβ signaling plays a pivotal role in tumor invasion [68], [69]. Our results indicates that Smad3, but not Smad2 (Figures S2, S3) is phosphorylated and translocated to the nucleus upon short term (3–6 h) incubation of the cells under hypoxic conditions and that this event persists for up to 16 h. Such activation of Smad3 seems to be a key mechanism by which hypoxia enhanced invadopodia formation since full inhibition of the percentage of cells forming invadopodia was observed in the presence of selective inhibitors of Smad3 phosphorylation/signaling as well as inhibitors of the TGFβΡ1 κινασε. It was reported that the cellular response to hypoxia involves signaling via Smad proteins. Indeed, brief exposure of HUVECs to hypoxia was shown to be sufficient to induce Smad phosphorylation through bioactivation of latent TGFβ [3]. There is now ample evidence that Smad2 and Smad3 have distinct and non-overlapping roles in TGFβ signaling and that these differ in cell types [70]. Interestingly, Smad3 has been linked to TGFβ-mediated EMT [71], an important event associated with cell migration and invasion [72]–[74]. In addition, Smad3 has been shown to be an important contributor to breast cancer cell invasion by controlling the expression of MMP-2 and MMP-9 [75], two metalloproteinases involved in matrix degradation and invadopodia functions [76], [77]. In contrast, Smad2 was shown to be required for TGFβ-induced growth inhibition in various cellular contexts [78], [79]. Therefore such strategic activation of Smad 3 under hypoxia might be important for cancer cell transformation into a more aggressive, invadopodia-forming phenotype.

Little is known concerning the selective activation of Smad2 versus Smad3, but this could occur at the level of phosphorylation, gene expression, protein stability, nuclear import/export mechanisms or cytoplasmic/plasma membrane sequestration by retention proteins in a cell-dependent and/or context-dependent fashion. We observed that even though Smad3 protein expression levels remained unchanged, hypoxia consistently and repetitively triggered an increase in Smad3 activation suggesting that hypoxia does not modify Smad3 stability or gene expression. Microtubule binding to Smads has been proposed to regulate TGFβ activity by sequestering and controlling the association and phosphorylation of Smads by activated TGFβ-receptor I [33]. It was recently reported that TGFβ-induced EMT was regulated by HDAC6, a cytoplasmic histone deacetylase that controls acetylated-level of α-tubulin and was shown to promote the activation of Smad3 in response to TGFβ [27]. HDAC6-mediated tubulin deacetylation is known to decrease tubulin stability and a recent study indicated that the structural integrity of β–tubulin played an essential role in mediating its interaction with Smad3 [80]. Here we show that selective inhibition of HDAC6 tubulin deacetylase activity restrained Smad3 nuclear accumulation following TGFβ stimulation or incubation under hypoxia, further suggesting the role of HDAC6 in the regulation of Smad3 activity in different cell context. We also demonstrated that inhibition or knockdown of HDAC6 resulted in a complete impairment of hypoxia-induced invadopodia formation, strengthening the role of Smad3 signaling pathway in invadopodia formation. Although deacetylation of α-tubulin by HDAC6 and subsequent Smad3 activation seems to be an important event in the induction of invadopodia production in hypoxia, other data link HDAC6 to tumor progression and cancer cell invasion through deacetylation of other cytoplasmic substrates. Indeed, it has been reported that inhibition of HDAC6 leads to hypoacetylation of HSP90αlpha, resulting in a diminution of MMP-2 maturation and decreased cell invasion [81]. An additional study has shown that HDAC6 regulates cell migration by altering acetylation of cortactin, an important component of invadopodia machinery [30] and that HDAC6 was required for invadopodia activity by regulating acetylated level of tubulin and cortactin [28]. Therefore, these observations suggest that other complementary mechanisms are involved leading to enhanced cell invasion under hypoxia.

Despite growing evidence indicating a role for HDAC6 in cancer invasion, inducers of HDAC6 activity are still poorly characterized. Among the few factors, TGFβ has been shown to induce HDAC6 activity through Smad3 activation in lung epithelial cells [27] and we showed here that stimulation of fibrosarcoma cells with TGFβ enhanced HDAC6 activity. Even though TGFβ has the capacity to activate HDAC6 in different cell systems, including ours, it is unlikely that endogenous TGFβ is the main factor involved in the early activation of HDAC6 in response to hypoxia. In fact, our results showed that short exposure of the cells to hypoxia (2–4 h) was sufficient to enhance HDAC6 deacetylase activity, while maximal activation/phosphorylation of Smad3 in response to hypoxia occurred at 6 h. In addition, inhibition of the TGFβR1 kinase did not reduce the enhanced HDAC6 action triggered by hypoxia, further suggesting the implication of other mediators/signaling pathways. Accordingly, a recent report has indicated that EGF-induced ERK-MAP kinase pathway enhanced cell migration by inducing deacetylation of tubulin through activation of HDAC6 [35]. EGFR is known to be activated under hypoxia [82], [83] and EGF is a well-known growth factor involved in cancer progression. Results presented here showed that hypoxia triggered EGFR phosphorylation and that incubation of the cells with EGF induced both HDAC6 activation and invadopodia formation (Figure S4). Furthermore, inhibition of the EGFR pathway strongly reduced hypodia-induced HDAC6 deacetylase activity, whereas parallel inhibition of TGFβ signaling had no effect. These results provided evidence that the early activation of HDAC6 under hypoxia was triggered by the activation of the EGFR pathway.

There is increasing evidence that EGF signaling acts in concert with Smads to regulate TGFβ responsive genes. EGF signaling through ERK1/2 was shown to promote the phosphorylation of R-Smads, resulting in down regulation of their activity and translocation to the nucleus [84], [85] while a recent study revealed that activation of the EGFR-ERK1/2 pathway resulted in the phosphorylation of Smad3 on its linker region leading to increased transcriptional activity [86]. Results presented here showed that EGF induces a small but significant increase in Smad3 activation/nuclear accumulation and amplified TGFβ-induced nuclear accumulation of Smad3 (Figure 7). Although it is possible that EGFR directly activates Smad3, our results indicating that EGFR is activated by hypoxia and that inhibition of EGFR strongly reduced hypoxia-induced HDAC6 deacetylase activity are consistent with a mechanism whereby EGFR activates HDAC6, which in turn induces tubulin deacetylase activity that facilitates Smad3 activation and nuclear translocation.

Hypoxia appeared to enhance HDAC6 activity through post-translational mechanisms because total HDAC6 protein levels were not altered by exposure of the cells to hypoxia. Recent data have revealed that HDAC6 activity can be regulated through phosphorylation. HDAC6 possesses several putative EGFR/MAPK phosphorylation sites and phosphorylation on Tyr570 by EGF stimulation has been shown to regulate HDAC6 tubulin deacetylase activity [87]. In addition, casein-kinase II (CK-II) was shown to phosphorylates HDAC6 on Ser458, causing activation of HDAC6 and regulation of aggresome formation in response to stress [88]. Interestingly, both hypoxia and EGF can up-regulate the activity of CK-II through phosphorylation or enhanced expression of the β-subunit [89], suggesting that activation of HDAC6 under hypoxia could be modulated by EGFR and/or CK-II. Future work would be needed to define the phosphorylation status of HDAC6 under hypoxia and to identify the nature of the protein kinases and phosphorylation sites involved.

A growing body of evidence indicates that the hypoxic microenvironment of solid tumors is a crucial inducer of cell invasion and metastasis. Consequently much interest has been drawn on “targetable” proteins and mechanisms to inhibit hypoxia-induced cell invasion. Hypoxia has previously been shown to induce HDAC6 gene and protein expression [31], [90] and here we show that this microenvironment also enhances HDAC6 activity, further strengthening the role of HDAC6 in hypoxia-induced cellular events. We also demonstrate that HDAC6 plays an essential role in hypoxia-induced invadopodia formation and cell invasion through selective activation of Smad3. Several small molecule inhibitors of HDAC6 are being developed and tested in vivo [91], [92] so the identification of HDAC6 as a key participant of hypoxia-induced cell invasion may provide a new and testable target to counter hypoxia-driven cell invasion and metastasis. It might also provide an alternative way to interfere with selective aspects of TGFβ signaling in hypoxic cells.

## Supporting Information

Figure S1 **Hypoxia induces the phosphorylation of Smad3.** HT-1080 cells were incubated in normoxia with or without TGFβ (0.5 ng/mL) and in hypoxia for 3 or 6 h. Total cell lysates were immunoblotted for p-Smad2, p-Smad3 and total Smad3.
(TIFF)

Figure S2 **Hypoxia does not cause p-Smad2 nuclear translocation.** HT-1080 cells were cultured on gelatin coated-slides and incubated for 3 and 6 hours in normoxia or hypoxia. Percentage of colocalization of p-Smad2 and p-Smad3 with the nucleus as described under Materials and Methods. Column, mean; bars, SEM, *** p<0.001.
(TIFF)

Figure S3 **Inhibition of HDACs influences invadopodia formation.** HT-1080 cells were cultured on fluorescent gelatin-coated slides and incubated in normoxia or hypoxia for 10 hours in the presence or absence of A) TSA or B) VPA at the indicated concentrations. The graphs show the percentage of cells forming invadopodia. Column, mean; bars, SEM; * p<0.05, ***p<0.001.
(TIFF)

Figure S4 **EGF induces invadopodia formation.** HT-1080 cells were cultured on fluorescent gelatin-coated slides in normoxia or hypoxia for 10 hours and treated with EGF (10 ng/mL, 20 ng/mL). The graph shows the percentage of cells forming invaopodia. Column, mean; bars, SEM; **; p = 0.01; *** p = 0.001.
(TIFF)

## References

[1] Krishnamachary B, Berg-Dixon S, Kelly B, Agani F, Feldser D, et al (2003) Regulation of colon carcinoma cell invasion by hypoxia-inducible factor 1. Cancer Res 63: 1138–1143. 12615733

[2] McMahon S, Grondin F, McDonald PP, Richard DE, Dubois CM (2005) Hypoxia-enhanced expression of the proprotein convertase furin is mediated by hypoxia-inducible factor-1: impact on the bioactivation of proproteins. J Biol Chem 280: 6561–6569. 15611046 10.1074/jbc.M413248200

[3] Zhang H, Akman HO, Smith EL, Zhao J, Murphy-Ullrich JE, et al (2003) Cellular response to hypoxia involves signaling via Smad proteins. Blood 101: 2253–2260. 12411310 10.1182/blood-2002-02-0629

[4] Akman HO, Zhang H, Siddiqui MA, Solomon W, Smith EL, et al (2001) Response to hypoxia involves transforming growth factor-beta2 and Smad proteins in human endothelial cells. Blood 98: 3324–3331. 11719370 10.1182/blood.v98.12.3324

[5] Shi YF, Fong CC, Zhang Q, Cheung PY, Tzang CH, et al (2007) Hypoxia induces the activation of human hepatic stellate cells LX-2 through TGF-beta signaling pathway. FEBS Lett 581: 203–210. 17187782 10.1016/j.febslet.2006.12.010

[6] Yoon SO, Shin S, Mercurio AM (2005) Hypoxia stimulates carcinoma invasion by stabilizing microtubules and promoting the Rab11 trafficking of the alpha6beta4 integrin. Cancer Res 65: 2761–2769. 15805276 10.1158/0008-5472.CAN-04-4122

[7] Arsenault D, Lucien F, Dubois CM (2011) Hypoxia enhances cancer cell invasion through relocalization of the proprotein convertase furin from the trans-Golgi network to the cell surface. J Cell Physiol. 10.1002/jcp.22792 21503879

[8] Munoz-Najar UM, Neurath KM, Vumbaca F, Claffey KP (2006) Hypoxia stimulates breast carcinoma cell invasion through MT1-MMP and MMP-2 activation. Oncogene 25: 2379–2392. 16369494 10.1038/sj.onc.1209273

[9] Lin HH, Li X, Chen JL, Sun X, Cooper FN, et al (2012) Identification of an AAA ATPase VPS4B-dependent pathway that modulates epidermal growth factor receptor abundance and signaling during hypoxia. Mol Cell Biol 32: 1124–1138. 22252323 10.1128/MCB.06053-11 PMC3295017

[10] Lucien F, Brochu-Gaudreau K, Arsenault D, Harper K, Dubois CM (2011) Hypoxia-induced invadopodia formation involves activation of NHE-1 by the p90 ribosomal S6 kinase (p90RSK). PLoS One 6: e28851. 22216126 10.1371/journal.pone.0028851 PMC3246449

[11] Park SY, Jun JA, Jeong KJ, Heo HJ, Sohn JS, et al (2011) Histone deacetylases 1, 6 and 8 are critical for invasion in breast cancer. Oncol Rep 25: 1677–1681. 21455583 10.3892/or.2011.1236

[12] Ahmad M, Hamid A, Hussain A, Majeed R, Qurishi Y, et al.. (2012) Understanding Histone Deacetylases in the Cancer Development and Treatment: An Epigenetic Perspective of Cancer Chemotherapy. DNA Cell Biol. 10.1089/dna.2011.1575 22462686

[13] Grozinger CM, Hassig CA, Schreiber SL (1999) Three proteins define a class of human histone deacetylases related to yeast Hda1p. Proc Natl Acad Sci U S A 96: 4868–4873. 10220385 10.1073/pnas.96.9.4868 PMC21783

[14] Marks P, Rifkind RA, Richon VM, Breslow R, Miller T, et al (2001) Histone deacetylases and cancer: causes and therapies. Nat Rev Cancer 1: 194–202. 11902574 10.1038/35106079

[15] Kim HJ, Bae SC (2011) Histone deacetylase inhibitors: molecular mechanisms of action and clinical trials as anti-cancer drugs. Am J Transl Res 3: 166–179. 21416059 PMC3056563

[16] Fukuda H, Sano N, Muto S, Horikoshi M (2006) Simple histone acetylation plays a complex role in the regulation of gene expression. Brief Funct Genomic Proteomic 5: 190–208. 16980317 10.1093/bfgp/ell032

[17] Lin HY, Chen CS, Lin SP, Weng JR, Chen CS (2006) Targeting histone deacetylase in cancer therapy. Med Res Rev 26: 397–413. 16450343 10.1002/med.20056

[18] Hess-Stumpp H (2005) Histone deacetylase inhibitors and cancer: from cell biology to the clinic. Eur J Cell Biol 84: 109–121. 15819394 10.1016/j.ejcb.2004.12.010

[19] Duvic M, Talpur R, Ni X, Zhang C, Hazarika P, et al (2007) Phase 2 trial of oral vorinostat (suberoylanilide hydroxamic acid, SAHA) for refractory cutaneous T-cell lymphoma (CTCL). Blood 109: 31–39. 16960145 10.1182/blood-2006-06-025999 PMC1785068

[20] Modesitt SC, Sill M, Hoffman JS, Bender DP (2008) A phase II study of vorinostat in the treatment of persistent or recurrent epithelial ovarian or primary peritoneal carcinoma: a Gynecologic Oncology Group study. Gynecol Oncol 109: 182–186. 18295319 10.1016/j.ygyno.2008.01.009

[21] Luu TH, Morgan RJ, Leong L, Lim D, McNamara M, et al (2008) A phase II trial of vorinostat (suberoylanilide hydroxamic acid) in metastatic breast cancer: a California Cancer Consortium study. Clin Cancer Res 14: 7138–7142. 18981013 10.1158/1078-0432.CCR-08-0122 PMC3543872

[22] Bruserud O, Stapnes C, Ersvaer E, Gjertsen BT, Ryningen A (2007) Histone deacetylase inhibitors in cancer treatment: a review of the clinical toxicity and the modulation of gene expression in cancer cell. Curr Pharm Biotechnol 8: 388–400. 18289048 10.2174/138920107783018417

[23] Bazzaro M, Lin Z, Santillan A, Lee MK, Wang MC, et al (2008) Ubiquitin proteasome system stress underlies synergistic killing of ovarian cancer cells by bortezomib and a novel HDAC6 inhibitor. Clin Cancer Res 14: 7340–7347. 19010849 10.1158/1078-0432.CCR-08-0642 PMC2744414

[24] Lee YS, Lim KH, Guo X, Kawaguchi Y, Gao Y, et al (2008) The cytoplasmic deacetylase HDAC6 is required for efficient oncogenic tumorigenesis. Cancer Res 68: 7561–7569. 18794144 10.1158/0008-5472.CAN-08-0188 PMC2978070

[25] Sakuma T, Uzawa K, Onda T, Shiiba M, Yokoe H, et al (2006) Aberrant expression of histone deacetylase 6 in oral squamous cell carcinoma. Int J Oncol 29: 117–124. 16773191

[26] Bradbury CA, Khanim FL, Hayden R, Bunce CM, White DA, et al (2005) Histone deacetylases in acute myeloid leukaemia show a distinctive pattern of expression that changes selectively in response to deacetylase inhibitors. Leukemia 19: 1751–1759. 16121216 10.1038/sj.leu.2403910

[27] Shan B, Yao TP, Nguyen HT, Zhuo Y, Levy DR, et al (2008) Requirement of HDAC6 for transforming growth factor-beta1-induced epithelial-mesenchymal transition. J Biol Chem 283: 21065–21073. 18499657 10.1074/jbc.M802786200 PMC2475688

[28] Rey M, Irondelle M, Waharte F, Lizarraga F, Chavrier P (2011) HDAC6 is required for invadopodia activity and invasion by breast tumor cells. Eur J Cell Biol 90: 128–135. 20970878 10.1016/j.ejcb.2010.09.004

[29] Tsunoda K, Oikawa H, Tada H, Tatemichi Y, Muraoka S, et al.. (2011) Nucleus Accumbens-Associated 1 Contributes to Cortactin Deacetylation and Augments the Migration of Melanoma Cells. J Invest Dermatol. 10.1038/jid.2011.110 21562571

[30] Zhang X, Yuan Z, Zhang Y, Yong S, Salas-Burgos A, et al (2007) HDAC6 modulates cell motility by altering the acetylation level of cortactin. Mol Cell 27: 197–213. 17643370 10.1016/j.molcel.2007.05.033 PMC2684874

[31] Kaluza D, Kroll J, Gesierich S, Yao TP, Boon RA, et al (2011) Class IIb HDAC6 regulates endothelial cell migration and angiogenesis by deacetylation of cortactin. EMBO J 30: 4142–4156. 21847094 10.1038/emboj.2011.298 PMC3199386

[32] Nogales E (2000) Structural insights into microtubule function. Annu Rev Biochem 69: 277–302. 10966460 10.1146/annurev.biochem.69.1.277

[33] Dong C, Li Z, Alvarez R Jr, Feng XH, Goldschmidt-Clermont PJ (2000) Microtubule binding to Smads may regulate TGF beta activity. Mol Cell 5: 27–34. 10678166 10.1016/s1097-2765(00)80400-1

[34] Inoue A, Yoshida N, Omoto Y, Oguchi S, Yamori T, et al (2002) Development of cDNA microarray for expression profiling of estrogen-responsive genes. J Mol Endocrinol 29: 175–192. 12370120 10.1677/jme.0.0290175

[35] Wang J, Lin A, Lu L (2010) Effect of EGF-induced HDAC6 activation on corneal epithelial wound healing. Invest Ophthalmol Vis Sci 51: 2943–2948. 20089874 10.1167/iovs.09-4639 PMC2891458

[36] Artym VV, Zhang Y, Seillier-Moiseiwitsch F, Yamada KM, Mueller SC (2006) Dynamic interactions of cortactin and membrane type 1 matrix metalloproteinase at invadopodia: defining the stages of invadopodia formation and function. Cancer Res 66: 3034–3043. 16540652 10.1158/0008-5472.CAN-05-2177

[37] Baldassarre M, Pompeo A, Beznoussenko G, Castaldi C, Cortellino S, et al (2003) Dynamin participates in focal extracellular matrix degradation by invasive cells. Mol Biol Cell 14: 1074–1084. 12631724 10.1091/mbc.E02-05-0308 PMC151580

[38] Lizarraga F, Poincloux R, Romao M, Montagnac G, Le Dez G, et al (2009) Diaphanous-related formins are required for invadopodia formation and invasion of breast tumor cells. Cancer Res 69: 2792–2800. 19276357 10.1158/0008-5472.CAN-08-3709

[39] Sakurai-Yageta M, Recchi C, Le Dez G, Sibarita JB, Daviet L, et al (2008) The interaction of IQGAP1 with the exocyst complex is required for tumor cell invasion downstream of Cdc42 and RhoA. J Cell Biol 181: 985–998. 18541705 10.1083/jcb.200709076 PMC2426946

[40] Wolf K, Wu YI, Liu Y, Geiger J, Tam E, et al (2007) Multi-step pericellular proteolysis controls the transition from individual to collective cancer cell invasion. Nat Cell Biol 9: 893–904. 17618273 10.1038/ncb1616

[41] Yamaguchi H, Lorenz M, Kempiak S, Sarmiento C, Coniglio S, et al (2005) Molecular mechanisms of invadopodium formation: the role of the N-WASP-Arp2/3 complex pathway and cofilin. J Cell Biol 168: 441–452. 15684033 10.1083/jcb.200407076 PMC2171731

[42] Kimura F, Iwaya K, Kawaguchi T, Kaise H, Yamada K, et al (2010) Epidermal growth factor-dependent enhancement of invasiveness of squamous cell carcinoma of the breast. Cancer Sci 101: 1133–1140. 20219074 10.1111/j.1349-7006.2010.01527.x PMC11158962

[43] Desmarais V, Yamaguchi H, Oser M, Soon L, Mouneimne G, et al (2009) N-WASP and cortactin are involved in invadopodium-dependent chemotaxis to EGF in breast tumor cells. Cell Motil Cytoskeleton 66: 303–316. 19373774 10.1002/cm.20361 PMC2747479

[44] Rottiers P, Saltel F, Daubon T, Chaigne-Delalande B, Tridon V, et al (2009) TGFbeta-induced endothelial podosomes mediate basement membrane collagen degradation in arterial vessels. J Cell Sci 122: 4311–4318. 19887587 10.1242/jcs.057448

[45] Mandal S, Johnson KR, Wheelock MJ (2008) TGF-beta induces formation of F-actin cores and matrix degradation in human breast cancer cells via distinct signaling pathways. Exp Cell Res 314: 3478–3493. 18848540 10.1016/j.yexcr.2008.09.013

[46] Baldassarre M, Ayala I, Beznoussenko G, Giacchetti G, Machesky LM, et al (2006) Actin dynamics at sites of extracellular matrix degradation. Eur J Cell Biol 85: 1217–1231. 17010475 10.1016/j.ejcb.2006.08.003

[47] Blanchette F, Rivard N, Rudd P, Grondin F, Attisano L, et al (2001) Cross-talk between the p42/p44 MAP kinase and Smad pathways in transforming growth factor beta 1-induced furin gene transactivation. J Biol Chem 276: 33986–33994. 11448947 10.1074/jbc.M100093200

[48] Wheelock MJ, Shintani Y, Maeda M, Fukumoto Y, Johnson KR (2008) Cadherin switching. J Cell Sci 121: 727–735. 18322269 10.1242/jcs.000455

[49] Shintani Y, Wheelock MJ, Johnson KR (2006) Phosphoinositide-3 kinase-Rac1-c-Jun NH2-terminal kinase signaling mediates collagen I-induced cell scattering and up-regulation of N-cadherin expression in mouse mammary epithelial cells. Mol Biol Cell 17: 2963–2975. 16624865 10.1091/mbc.E05-12-1123 PMC1483033

[50] Maeda M, Johnson KR, Wheelock MJ (2005) Cadherin switching: essential for behavioral but not morphological changes during an epithelium-to-mesenchyme transition. J Cell Sci 118: 873–887. 15713751 10.1242/jcs.01634

[51] Dubois CM, Blanchette F, Laprise MH, Leduc R, Grondin F, et al (2001) Evidence that furin is an authentic transforming growth factor-beta1-converting enzyme. Am J Pathol 158: 305–316. 11141505 10.1016/s0002-9440(10)63970-3 PMC1850265

[52] Cui Y, Jean F, Thomas G, Christian JL (1998) BMP-4 is proteolytically activated by furin and/or PC6 during vertebrate embryonic development. EMBO J 17: 4735–4743. 9707432 10.1093/emboj/17.16.4735 PMC1170802

[53] Jean F, Stella K, Thomas L, Liu G, Xiang Y, et al (1998) alpha1-Antitrypsin Portland, a bioengineered serpin highly selective for furin: application as an antipathogenic agent. Proc Natl Acad Sci U S A 95: 7293–7298. 9636142 10.1073/pnas.95.13.7293 PMC22594

[54] Feng XH, Derynck R (2005) Specificity and versatility in tgf-beta signaling through Smads. Annu Rev Cell Dev Biol 21: 659–693. 16212511 10.1146/annurev.cellbio.21.022404.142018

[55] Basu RK, Hubchak S, Hayashida T, Runyan CE, Schumacker PT, et al (2011) Interdependence of HIF-1alpha and TGF-beta/Smad3 signaling in normoxic and hypoxic renal epithelial cell collagen expression. Am J Physiol Renal Physiol 300: F898–905. 21209004 10.1152/ajprenal.00335.2010 PMC3075002

[56] Chae KS, Kang MJ, Lee JH, Ryu BK, Lee MG, et al (2011) Opposite functions of HIF-alpha isoforms in VEGF induction by TGF-beta1 under non-hypoxic conditions. Oncogene 30: 1213–1228. 21057546 10.1038/onc.2010.498

[57] Sawyer JS, Anderson BD, Beight DW, Campbell RM, Jones ML, et al (2003) Synthesis and activity of new aryl- and heteroaryl-substituted pyrazole inhibitors of the transforming growth factor-beta type I receptor kinase domain. J Med Chem 46: 3953–3956. 12954047 10.1021/jm0205705

[58] Jinnin M, Ihn H, Tamaki K (2006) Characterization of SIS3, a novel specific inhibitor of Smad3, and its effect on transforming growth factor-beta1-induced extracellular matrix expression. Mol Pharmacol 69: 597–607. 16288083 10.1124/mol.105.017483

[59] Hubbert C, Guardiola A, Shao R, Kawaguchi Y, Ito A, et al (2002) HDAC6 is a microtubule-associated deacetylase. Nature 417: 455–458. 12024216 10.1038/417455a

[60] Haggarty SJ, Koeller KM, Wong JC, Grozinger CM, Schreiber SL (2003) Domain-selective small-molecule inhibitor of histone deacetylase 6 (HDAC6)-mediated tubulin deacetylation. Proc Natl Acad Sci U S A 100: 4389–4394. 12677000 10.1073/pnas.0430973100 PMC153564

[61] Dubois CM, Laprise MH, Blanchette F, Gentry LE, Leduc R (1995) Processing of transforming growth factor beta 1 precursor by human furin convertase. J Biol Chem 270: 10618–10624. 7737999 10.1074/jbc.270.18.10618

[62] Scandura JM, Boccuni P, Massague J, Nimer SD (2004) Transforming growth factor beta-induced cell cycle arrest of human hematopoietic cells requires p57KIP2 up-regulation. Proc Natl Acad Sci U S A 101: 15231–15236. 15477587 10.1073/pnas.0406771101 PMC524079

[63] Krasagakis K, Tholke D, Farthmann B, Eberle J, Mansmann U, et al (1998) Elevated plasma levels of transforming growth factor (TGF)-beta1 and TGF-beta2 in patients with disseminated malignant melanoma. Br J Cancer 77: 1492–1494. 9652767 10.1038/bjc.1998.245 PMC2150189

[64] Ito N, Kawata S, Tamura S, Takaishi K, Shirai Y, et al (1991) Elevated levels of transforming growth factor beta messenger RNA and its polypeptide in human hepatocellular carcinoma. Cancer Res 51: 4080–4083. 1649698

[65] Behzadian MA, Wang XL, Al-Shabrawey M, Caldwell RB (1998) Effects of hypoxia on glial cell expression of angiogenesis-regulating factors VEGF and TGF-beta. Glia 24: 216–225. 9728767

[66] Sato H, Kinoshita T, Takino T, Nakayama K, Seiki M (1996) Activation of a recombinant membrane type 1-matrix metalloproteinase (MT1-MMP) by furin and its interaction with tissue inhibitor of metalloproteinases (TIMP)-2. FEBS Lett 393: 101–104. 8804434 10.1016/0014-5793(96)00861-7

[67] Yana I, Weiss SJ (2000) Regulation of membrane type-1 matrix metalloproteinase activation by proprotein convertases. Mol Biol Cell 11: 2387–2401. 10888676 10.1091/mbc.11.7.2387 PMC14927

[68] Akhurst RJ, Derynck R (2001) TGF-beta signaling in cancer–a double-edged sword. Trends Cell Biol 11: S44–51. 11684442 10.1016/s0962-8924(01)02130-4

[69] Massague J (2008) TGFbeta in Cancer. Cell 134: 215–230. 18662538 10.1016/j.cell.2008.07.001 PMC3512574

[70] Brown KA, Pietenpol JA, Moses HL (2007) A tale of two proteins: differential roles and regulation of Smad2 and Smad3 in TGF-beta signaling. J Cell Biochem 101: 9–33. 17340614 10.1002/jcb.21255

[71] Roberts AB, Tian F, Byfield SD, Stuelten C, Ooshima A, et al (2006) Smad3 is key to TGF-beta-mediated epithelial-to-mesenchymal transition, fibrosis, tumor suppression and metastasis. Cytokine Growth Factor Rev 17: 19–27. 16290023 10.1016/j.cytogfr.2005.09.008

[72] Deckers M, van Dinther M, Buijs J, Que I, Lowik C, et al (2006) The tumor suppressor Smad4 is required for transforming growth factor beta-induced epithelial to mesenchymal transition and bone metastasis of breast cancer cells. Cancer Res 66: 2202–2209. 16489022 10.1158/0008-5472.CAN-05-3560

[73] Valcourt U, Kowanetz M, Niimi H, Heldin CH, Moustakas A (2005) TGF-beta and the Smad signaling pathway support transcriptomic reprogramming during epithelial-mesenchymal cell transition. Mol Biol Cell 16: 1987–2002. 15689496 10.1091/mbc.E04-08-0658 PMC1073677

[74] Piek E, Moustakas A, Kurisaki A, Heldin CH, ten Dijke P (1999) TGF-(beta) type I receptor/ALK-5 and Smad proteins mediate epithelial to mesenchymal transdifferentiation in NMuMG breast epithelial cells. J Cell Sci 112 ( Pt 24): 4557–4568. 10.1242/jcs.112.24.4557 10574705

[75] Wiercinska E, Naber HP, Pardali E, van der Pluijm G, van Dam H, et al.. (2010) The TGF-beta/Smad pathway induces breast cancer cell invasion through the up-regulation of matrix metalloproteinase 2 and 9 in a spheroid invasion model system. Breast Cancer Res Treat. 10.1007/s10549-010-1147-x 20821046

[76] Nascimento CF, Gama-De-Souza LN, Freitas VM, Jaeger RG (2010) Role of MMP9 on invadopodia formation in cells from adenoid cystic carcinoma. Study by laser scanning confocal microscopy. Microsc Res Tech 73: 99–108. 19658178 10.1002/jemt.20761

[77] Chen WT, Wang JY (1999) Specialized surface protrusions of invasive cells, invadopodia and lamellipodia, have differential MT1-MMP, MMP-2, and TIMP-2 localization. Ann N Y Acad Sci 878: 361–371. 10415741 10.1111/j.1749-6632.1999.tb07695.x

[78] Stegmuller J, Huynh MA, Yuan Z, Konishi Y, Bonni A (2008) TGFbeta-Smad2 signaling regulates the Cdh1-APC/SnoN pathway of axonal morphogenesis. J Neurosci 28: 1961–1969. 18287512 10.1523/JNEUROSCI.3061-07.2008 PMC6671436

[79] Ju W, Ogawa A, Heyer J, Nierhof D, Yu L, et al (2006) Deletion of Smad2 in mouse liver reveals novel functions in hepatocyte growth and differentiation. Mol Cell Biol 26: 654–667. 16382155 10.1128/MCB.26.2.654-667.2006 PMC1346892

[80] Gong K, Xing D, Li P, Hilgers RH, Hage FG, et al (2011) cGMP inhibits TGF-beta signaling by sequestering Smad3 with cytosolic beta2-tubulin in pulmonary artery smooth muscle cells. Mol Endocrinol 25: 1794–1803. 21868450 10.1210/me.2011-1009 PMC3182417

[81] Yang Y, Rao R, Shen J, Tang Y, Fiskus W, et al (2008) Role of acetylation and extracellular location of heat shock protein 90alpha in tumor cell invasion. Cancer Res 68: 4833–4842. 18559531 10.1158/0008-5472.CAN-08-0644 PMC2665713

[82] Mishra OP, Ashraf QM, Delivoria-Papadopoulos M (2010) Hypoxia-induced activation of epidermal growth factor receptor (EGFR) kinase in the cerebral cortex of newborn piglets: the role of nitric oxide. Neurochem Res 35: 1471–1477. 20532621 10.1007/s11064-010-0208-1

[83] Wang X, Schneider A (2010) HIF-2alpha-mediated activation of the epidermal growth factor receptor potentiates head and neck cancer cell migration in response to hypoxia. Carcinogenesis 31: 1202–1210. 20395290 10.1093/carcin/bgq078 PMC2893799

[84] Kretzschmar M, Doody J, Massague J (1997) Opposing BMP and EGF signalling pathways converge on the TGF-beta family mediator Smad1. Nature 389: 618–622. 9335504 10.1038/39348

[85] Kretzschmar M, Doody J, Timokhina I, Massague J (1999) A mechanism of repression of TGFbeta/ Smad signaling by oncogenic Ras. Genes Dev 13: 804–816. 10197981 10.1101/gad.13.7.804 PMC316599

[86] Sasseville M, Ritter LJ, Nguyen TM, Liu F, Mottershead DG, et al (2010) Growth differentiation factor 9 signaling requires ERK1/2 activity in mouse granulosa and cumulus cells. J Cell Sci 123: 3166–3176. 20736313 10.1242/jcs.063834

[87] Deribe YL, Wild P, Chandrashaker A, Curak J, Schmidt MH, et al (2009) Regulation of epidermal growth factor receptor trafficking by lysine deacetylase HDAC6. Sci Signal 2: ra84. 20029029 10.1126/scisignal.2000576

[88] Watabe M, Nakaki T (2011) Protein kinase CK2 regulates the formation and clearance of aggresomes in response to stress. J Cell Sci 124: 1519–1532. 21486957 10.1242/jcs.081778

[89] Mottet D, Ruys SP, Demazy C, Raes M, Michiels C (2005) Role for casein kinase 2 in the regulation of HIF-1 activity. Int J Cancer 117: 764–774. 15957168 10.1002/ijc.21268

[90] Wang S, Li X, Parra M, Verdin E, Bassel-Duby R, et al (2008) Control of endothelial cell proliferation and migration by VEGF signaling to histone deacetylase 7. Proc Natl Acad Sci U S A 105: 7738–7743. 18509061 10.1073/pnas.0802857105 PMC2409381

[91] Dallavalle S, Pisano C, Zunino F (2012) Development and therapeutic impact of HDAC6-selective inhibitors. Biochem Pharmacol 84: 756–765. 22728920 10.1016/j.bcp.2012.06.014

[92] Bennewith KL, Dedhar S (2011) Targeting hypoxic tumour cells to overcome metastasis. BMC Cancer 11: 504. 22128892 10.1186/1471-2407-11-504 PMC3247198

