# Supplementary figures and images for: HDAC6 Deacetylase Activity Is Required for Hypoxia-Induced Invadopodia Formation and Cell Invasion
Source: PLoS One. 2013 Feb 6;8(2):e55529. doi: 10.1371/journal.pone.0055529 (PMC3566011; doi:10.1371/journal.pone.0055529)

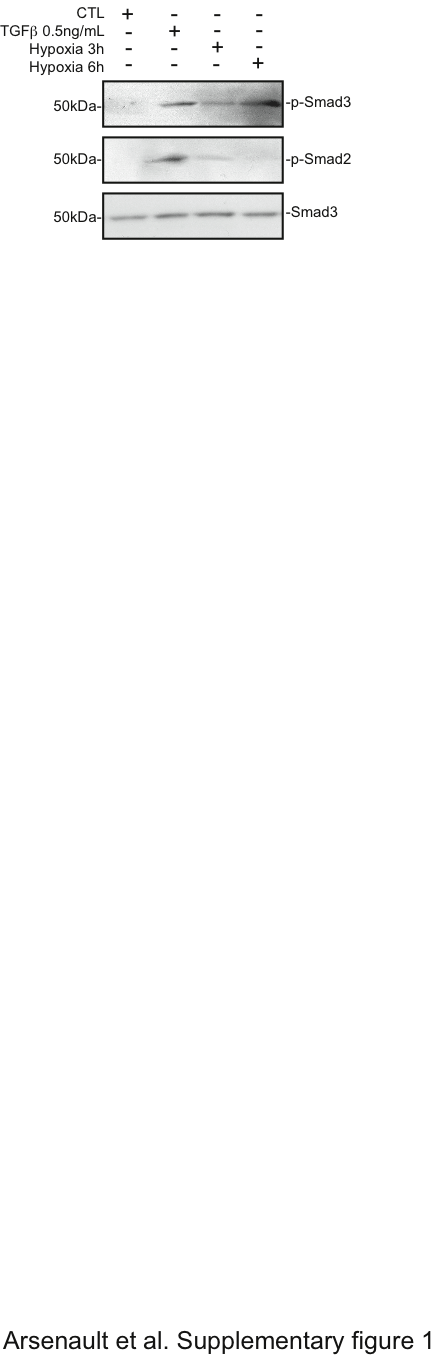

Supplement: Figure S1 — Hypoxia induces the phosphorylation of Smad3. HT-1080 cells were incubated in normoxia with or without TGFβ (0.5 ng/mL) and in hypoxia for 3 or 6 h. Total cell lysates were immunoblotted for p-Smad2, p-Smad3 and total Smad3. (TIFF) [file pone.0055529.s001.tiff]

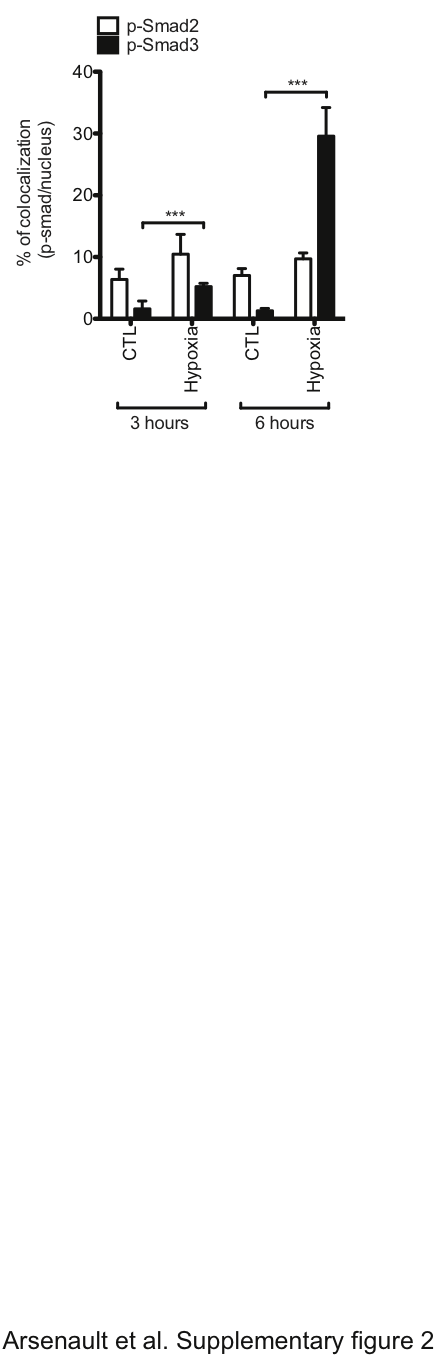

Supplement: Figure S2 — Hypoxia does not cause p-Smad2 nuclear translocation. HT-1080 cells were cultured on gelatin coated-slides and incubated for 3 and 6 hours in normoxia or hypoxia. Percentage of colocalization of p-Smad2 and p-Smad3 with the nucleus as described under Materials and Methods. Column, mean; bars, SEM, *** p<0.001. (TIFF) [file pone.0055529.s002.tiff]

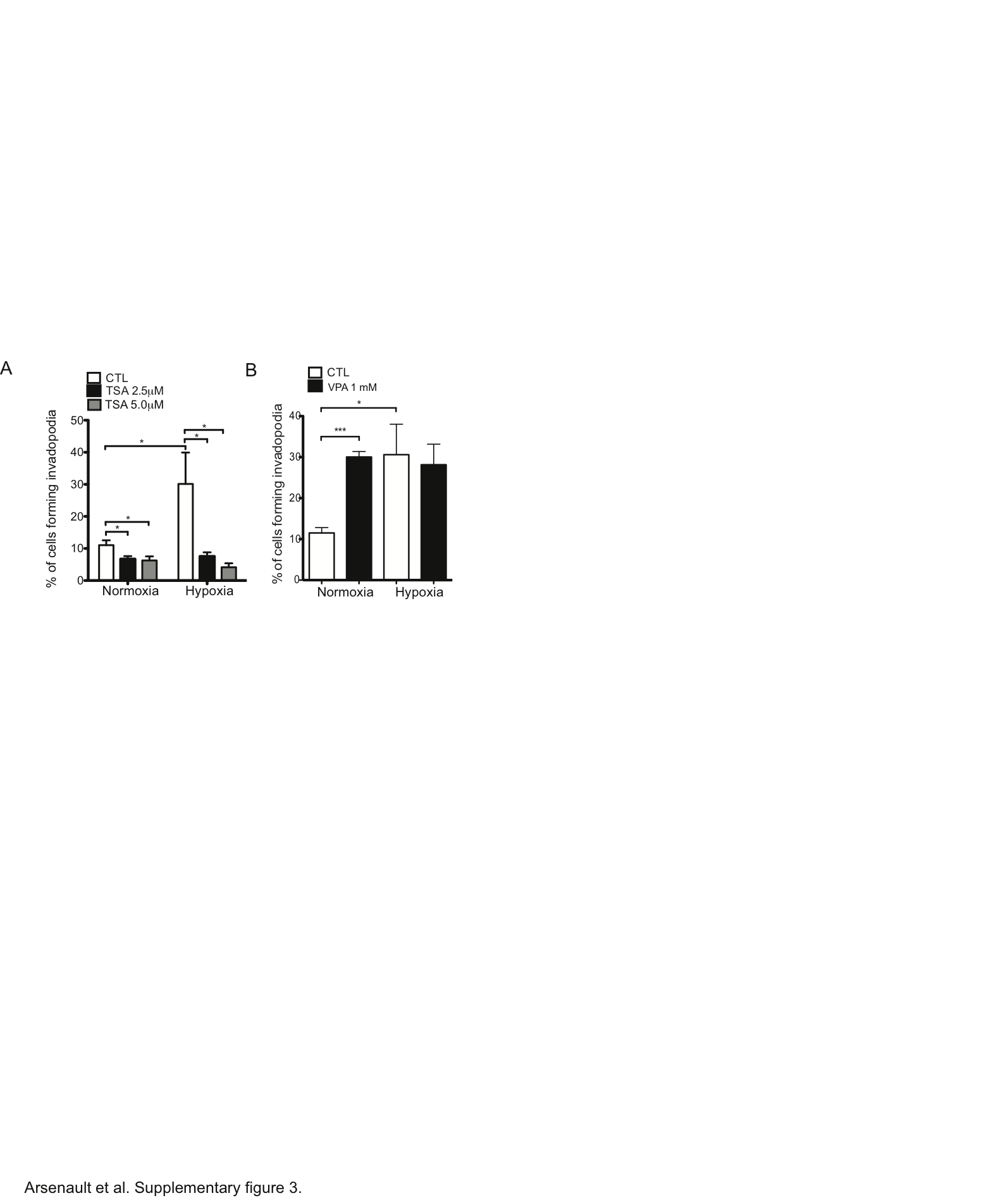

Supplement: Figure S3 — Inhibition of HDACs influences invadopodia formation. HT-1080 cells were cultured on fluorescent gelatin-coated slides and incubated in normoxia or hypoxia for 10 hours in the presence or absence of A) TSA or B) VPA at the indicated concentrations. The graphs show the percentage of cells forming invadopodia. Column, mean; bars, SEM; * p<0.05, ***p<0.001. (TIFF) [file pone.0055529.s003.tiff]

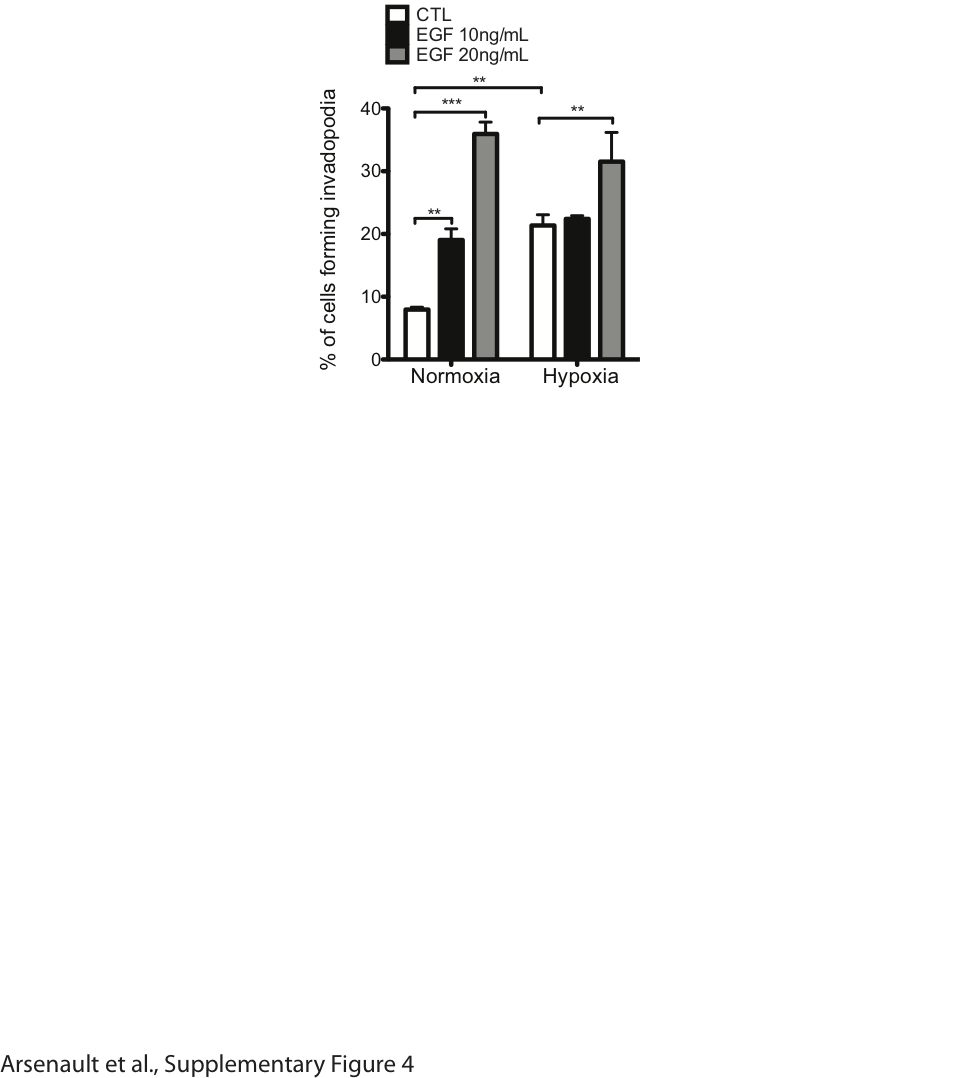

Supplement: Figure S4 — EGF induces invadopodia formation. HT-1080 cells were cultured on fluorescent gelatin-coated slides in normoxia or hypoxia for 10 hours and treated with EGF (10 ng/mL, 20 ng/mL). The graph shows the percentage of cells forming invaopodia. Column, mean; bars, SEM; **; p = 0.01; *** p = 0.001. (TIFF) [file pone.0055529.s004.tiff]
